# Printed Composite Film with Microporous/Micropyramid Hybrid Conductive Architecture for Multifunctional Flexible Force Sensors

**DOI:** 10.3390/nano14010063

**Published:** 2023-12-25

**Authors:** Yi-Fei Wang, Junya Yoshida, Yasunori Takeda, Ayako Yoshida, Takeru Kaneko, Tomohito Sekine, Daisuke Kumaki, Shizuo Tokito

**Affiliations:** Research Center for Organic Electronics (ROEL), Yamagata University, 4-3-16, Jonan, Yonezawa 992-8510, Yamagata, Japan; tww84653@st.yamagata-u.ac.jp (J.Y.); y.takeda@yz.yamagata-u.ac.jp (Y.T.); ayako_yoshida@yz.yamagata-u.ac.jp (A.Y.); t221438m@st.yamagata-u.ac.jp (T.K.); tomohito@yz.yamagata-u.ac.jp (T.S.); d_kumaki@yz.yamagata-u.ac.jp (D.K.)

**Keywords:** printed flexible sensors, conductive composite, porous structure, micropyramid structure, multifunctional force detection

## Abstract

Porous structures and micropatterning surfaces play a crucial role in the development of highly sensitive force sensors. However, achieving these two conductive architectures typically requires the synthesis of complex materials and expensive manufacturing processes. In this study, we introduce a novel conductive composite film featuring a microporous/micropyramid hybrid conductive architecture, which is achieved through a straightforward process of materials mixing and one-step screen printing. By utilizing a deep eutectic solvent in the ink component, micropores are induced in the printed composite, while the mesh of the screen mask acts as a template, resulting in a micropyramid film surface. We have successfully realized highly sensitive flexible force sensors (0.15 kPa^−1^) with multifunctional capabilities for perceiving normal force and shear force.

## 1. Introduction

Flexible sensors have emerged as a promising technology in the domains of wearable technology and robotics, offering diverse applications including human–machine interfaces, prosthetics, and robotic manipulation [1,2,3,4]. Among the various categories of flexible sensors, flexible force sensors have garnered significant attention due to their capacity to accurately detect and quantify applied forces, thereby providing real-time force feedback to enhance user experience and safety [5,6,7]. Conductive composite materials have displayed remarkable potential in the development of flexible pressure sensors, owing to their excellent electrical conductivity and mechanical flexibility [8,9]. These materials facilitate seamless integration into flexible sensor designs, enabling them to conform to irregular surfaces, withstand deformations, and deliver reliable and precise force measurements [10,11,12].

The sensitivity of pressure sensors is profoundly influenced by the morphology of the conductive film [13,14]. Two critical architectural features, microporous and micropatterned structures, play a pivotal role in enhancing pressure sensor sensitivity [15,16,17,18,19,20,21,22,23,24,25,26]. Microporous conductive structures induce a substantial change in bulk resistance when subjected to pressure, owing to their porous nature, which allows the elastic film to be easily compressed. Consequently, this results in heightened sensitivity and an extended sensing range [15,16,17,18,19,20,21,22]. Conversely, micropatterned structures are related to changes in contact resistance between the sensing layer and the electrode. The microstructure of the sensor mitigates abrupt on–off behavior due to gradually changing contact points, leading to an expanded working range [23,24,25,26]. However, the achievement of these desired microporous and micropatterned structures in conductive films presents significant challenges. Porous structures are typically realized through methods such as immersing porous materials like sponges in a conductive solution or employing a soluble template, such as sugar or salt, in a conductive polymer composite [19,20]. These techniques involve complex and time-consuming processes and present challenges for device patterning, often resulting in films with thicknesses on the order of several millimeters. Micropatterned surfaces are typically achieved through molding, which requires expensive templates [24,25]. More recently, natural-based patterning and laser abrasion have been explored, but they still necessitate multiple steps and often produce films of large dimensions that cannot be readily patterned on the target substrate [27,28]. These material and process limitations significantly hinder the practical application of these sensing structures.

This study introduces an innovative approach for fabricating a conductive film with a hybrid microporous/micropyramid architecture for flexible force sensors. This exceptional conductive architecture is achieved through the design of a composite ink and the selection of a fabrication method. We utilized a deep eutectic solvent (DES) as a liquid template in a mixture of PDMS and graphite composite, which enabled the creation of a porous structure through an annealing process. By adjusting the ink’s viscosity with a high graphite loading, we employed the screen-printing method with a mesh mask as the template during printing, resulting in a readily achievable micropyramid structure in the printed film. This proposed method offers advantages in terms of scalability, performance, and cost-effectiveness. We successfully realized high-performance pressure sensors and sensor arrays, demonstrating comprehensive capabilities in detecting pulse waves, human finger tapping, and shear forces.

## 2. Materials and Methods

### 2.1. Materials

Poly(ethylene-phthalate) (PEN) film (Q65HA, 100 μm thick) was procured from Teijin Dupont Film (Tokyo, Japan). PDMS (Sylgard 184) was sourced from Dow Corning (Midland, MI, USA). Benzophenone (BP) and Diphenylamine (DP) were obtained from TCI Chemical (Tokyo, Japan). Graphite powder (<20 μm) was purchased from Aldrich (Tokyo, Japan). Screen-printable Ag paste (XA-3797) was acquired from Fujikura Kasei (Tokyo, Japan), and the screen masks and stencil masks were obtained from SONOCOM Co., Ltd. (Tokyo, Japan). All the materials were utilized as received.

### 2.2. Ink Synthesis

The preparation of the printable composite inks was conducted in accordance with our prior publications [29,30,31], with minor adjustments. As depicted in Figure S1, BP and DP were blended in a 1:1 molar ratio to create a deep eutectic liquid. The resulting solid mixture was stored at room temperature for 2 h or annealed at 90 °C for 30 min to yield a clear yellowish liquid. A suitable quantity of graphite powder was introduced to the deep eutectic solvent (DES) and blended using a planetary centrifugal mixer (THINKY MIXER AR-100) for 7 min (5 min of mixing and 2 min of degassing). The obtained DES–graphite gel was further mixed with PDMS (base polymer: cure agent 10:1) using a mixer for 15 min to produce a slurry-like composite ink. In this investigation, the weight ratio of PDMS to DES was maintained at 1:1 in the final ink formulations. Detailed information on the ink formulations is provided in Table S1.

### 2.3. Sensor Fabrication

The sensors were fabricated using an automatic screen printer (Micro-tec Co., Ltd., Urayasu, Chiba, Japan). Initially, electrodes were printed onto the PEN film using silver paste and then sintered at 150 °C for 30 min. Subsequently, the composite ink was printed on another PEN substrate, and an annealing process was conducted at 75 °C for 1 h followed by 140 °C for 30 min to create the sensing layer. The sensing layer was cut into a circular shape using a knife and assembled to the electrodes in a face-to-face configuration. To ensure stability, a 3M telegram tape was employed to seal the device. The fabricated sensor was trimmed to the desired dimensions and affixed with an FPC connector for subsequent characterization and measurements.

### 2.4. Characterization

An optical microscope (Keyence VHX-7000, Keyence, Osaka, Japan) was utilized to capture optical images of the printed traces and substrate. Scanning electron microscopy (SEM) images were obtained using a HITACHI tabletop microscope (TM4000Plus, Hitachi High-Technologies Corp., Tokyo, Japan). Photographs and videos of the sensors in the application demonstration were captured with a smartphone camera. The pressure-sensing tests were performed using a force gauge with an auto stage (IMADA). Resistance measurements were recorded using a digital multimeter (KEITHLEY DMM6500, Tektronix Inc., Cedar Hills, OR, USA) with a 2-wire measurement setup. For the demonstration of human pulse wave and finger tape monitoring, the developed sensors were affixed to the hand of a male volunteer (34 years old). The experiments involving the monitoring of human pulse waves and finger tapping were conducted with the approval of the Yamagata University’s institutional review board (R05-16).

## 3. Results and Discussion

### 3.1. Sensor Design and Fabrication

**Figure 1:**
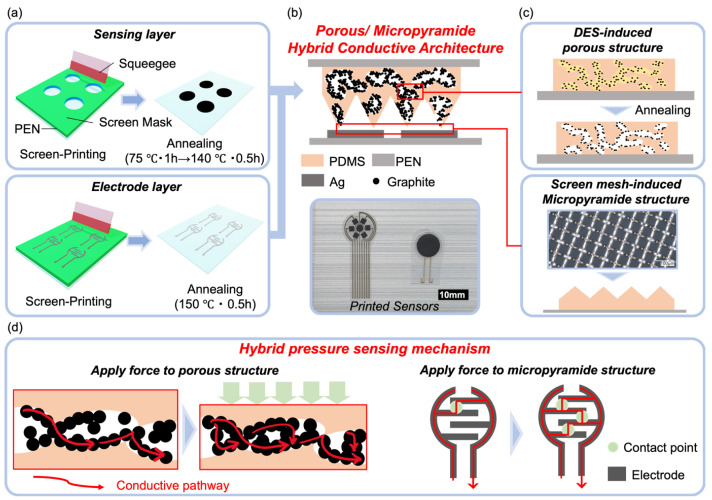
(**a**) Schematic of the fabrication process for the printed pressure sensor. (**b**) Illustration of the sensor structure showcasing the hybrid porous/micropyramid-conductive architecture and a photograph of the printed sensors. (**c**) Illustration of the formation mechanism of the sensing layer with the hybrid porous/micropyramid-conductive architecture. (**d**) Diagram illustrating the pressure sensing mechanism of the printed sensors.

Constructing a porous structure or a micropatterned surface for a conductive film are widely acknowledged as two effective approaches to achieve high-performance resistive-type pressure sensors. However, methods for realizing such conductive structures, let alone a hybrid structure combining both architectures, have posed significant challenges. Researchers have intensively explored the use of sugar templates (or salt particles) to create porous structures, often without highlighting the difficulties associated with template removal, thin film formation, and patterning. Meanwhile, micropatterning surfaces typically involve multi-step molding processes with high costs, making them less conducive to scalable manufacturing. While these conventional methods may hold academic value, they fall short in promoting the practical application of flexible sensors, creating a gap between sensor performance and manufacturability. Differing from these previous methods, we have developed a one-step process that involves selecting the right material system and adjusting viscosity while utilizing a printing mask. The sensor layer is realized through a single-step screen printing and annealing process, as depicted in Figure 1a. This scalable approach, devoid of complex treatments, allows for the straightforward production of devices suitable for both mass production and well-defined patterning (see Figure S2). The printed sensing layer and the printed electrode layer are seamlessly assembled face-to-face and sealed using 3M tape, enabling us to easily create devices with both single sensor patterns and sensor arrays (Figure 1b).

The formation of the hybrid porous/pyramid structure can be attributed to the design of the material system and the selection of the printing process (Figure 1c). The ink is based on our previous discovery, wherein a deep eutectic solvent (DES) induces phase separation between carbon materials and PDMS, serving as a liquid template for the formation of a porous structure [28]. This ink system enables the realization of a porous structure with a one-step printing and annealing process. We use graphite as the conductive filler for this ink and have discovered that it can form a multiscale porous structure due to the porous nature of the graphite powder. As for the micropyramid surface morphology, we have innovatively designed a new approach that uses the screen mask to induce it. The screen mask includes microscale mesh, and by adjusting the ink viscosity, the micropyramid structures are raised by the screen mesh during the lift-off of the screen-printing mask. This ingenious design allows for the in situ formation of the micropyramid structure.

The expected working mechanism of the printed sensors is illustrated in Figure 1d, where the hybrid conductive structure induces a hybrid response mode. When pressure is applied to the sensor, the film’s pores are compressed, resulting in an increase in the conductive pathways for the graphite and, consequently, a decrease in resistance. Simultaneously, the contact points between the sensing layer and electrode layer increase due to the micropatterned structure, also leading to a decrease in resistance. The combined effect is expected to result in a high-performance sensor compared to those with only individual structures. Thus, in this work, we have developed a sensor that combines the advantages of both conductive structures but achieves this using a significantly simpler process than each of them individually. We believe that this design will have a profound impact on sensor development, benefiting both academia and industry alike.

### 3.2. Morphology Control of Printed Sensing Layers

**Figure 2:**
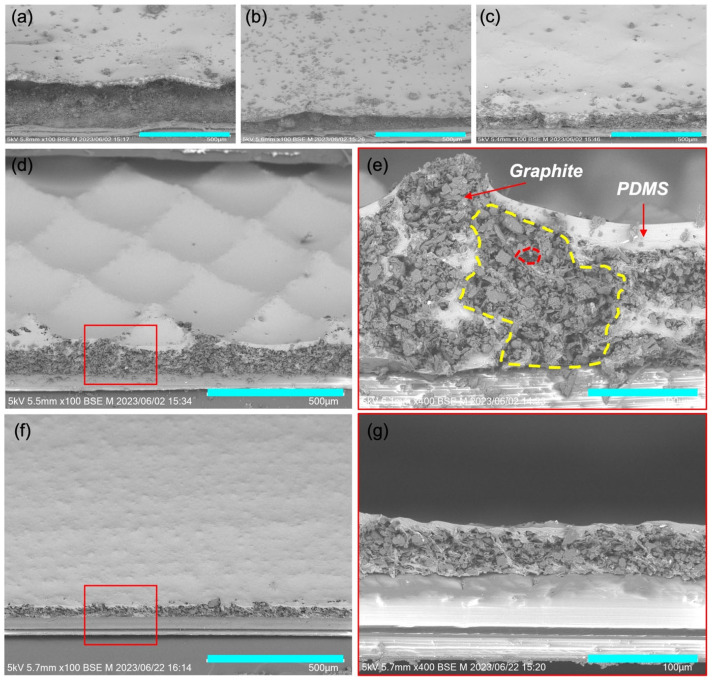
Surface morphologies of the printed composite films. (**a**–**c**) SEM images taken at a 45-degree observation angle of the screen-printed conductive films using (**a**) ink-1, (**b**) ink-2, (**c**) ink-3, and (**d**) ink-4. (**e**) Cross-sectional SEM image of the screen-printed conductive film using ink-4, highlighting the typical large pores marked by a yellow curve and small pores marked by a red curve. (**f**) SEM image at a 45-degree observation angle of the stencil-printed conductive films using ink-4. (**g**) Cross-sectional SEM image of the stencil-printed conductive film using ink-4. Scale bars: (**a**–**d**,**f**) 500 μm; (**e**,**g**) 100 μm. The red box in (**d**) and (**f**) indicates the position of films featured by (**e**) and (**g**), respectively.

**Figure 3:**
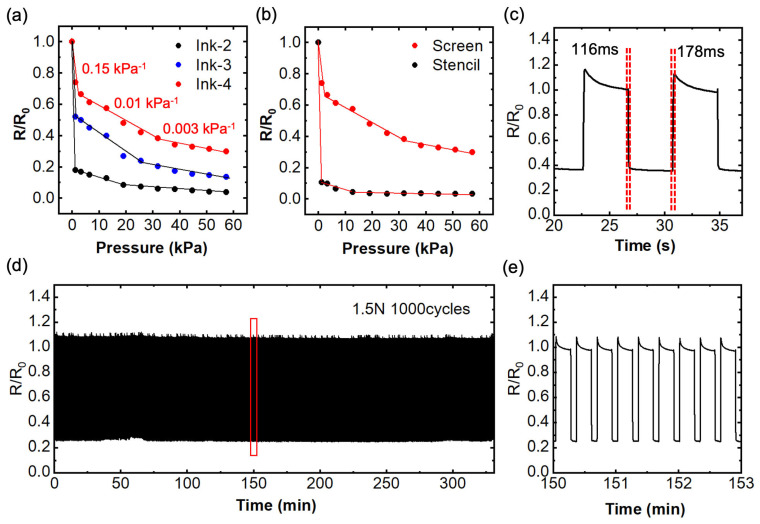
Electrical–mechanical performances of sensors with various composite films. (**a**) Relative resistance changes in screen-printed sensors using composite films of ink-2, ink-3, and ink-4 under varying applied pressures. (**b**) Relative resistance changes in sensors utilizing the composite film of ink-4, comparing screen printing and stencil printing under different applied pressures. (**c**) Response and recovery times of a screen-printed sensor based on ink-4. (**d**) Stability assessment of a screen-printed sensor based on ink-4 under a cyclic force of 1.5 N for 1000 cycles. (**e**) Enlarged image of the measurement results shown in the red frame of (**d**).

The scanning electron microscopy (SEM) images of the surface morphologies of the printed film are depicted in Figure 2. Within the developed ink formulations, the weight ratio of PDMS to DES was consistently maintained at 1:1. However, the mass loading of graphite was varied, with a weight ratio of graphite to PDMS of 0.5, 0.75, 1, and 1.25 for ink-1, ink-2, ink-3, and ink-4, respectively. As illustrated in Figure 2a–e, it is evident that as the graphite loading increases, the surface morphology becomes progressively more distinct. At lower loadings, the surface retains a flat appearance, primarily due to the leveling effect of the printed ink. In contrast, ink-4 exhibits a well-defined micropyramid structure with a height of 50 μm. Figure 3a presents the optical microscopy image of the screen mesh mask used in the printing process. Characterized by a mesh number of 100, the mask has a line distance of approximately 254 μm. The resulting printed composite film exhibits a distinctive micropyramid structure with a pitch size of approximately 250 μm, as illustrated in Figure S3b. Importantly, this pitch size perfectly aligns with the line distance of the screen mesh, strongly suggesting that the observed micropyramid structure in the printed composite film is induced by the screen mesh. We attribute this phenomenon to the high viscosity of ink-4, which contains a high loading of graphite. To enhance the understanding of the micropyramid structure formation process, we have included an illustration in Figure S4. As the ink is applied, it passes through the mesh onto the substrate, forming an initial ink film. Lifting off the screen mask causes the high-viscosity ink to be drawn upward along the mesh lines, resulting in the formation of a micropyramid shape in the vertical direction of the film. Additionally, the high viscosity of ink-4 plays a crucial role in maintaining the film’s shape during the annealing process, ensuring the preservation of the micropyramid structure. Continuing to increase the loading of graphite will result in excessively high viscosity, yielding a semi-solid ink (ink-5). Instead of being drawn upward to form a micropyramid structure, the ink film is imprinted by the screen mesh, resulting in a grid-like texture on the film’s surface (Figure S5). Consequently, ink-4 is defined as the optimal ink formulation.

In Figure 2e, a cross-section image of the printed film of ink-4 reveals a multiscale porous structure. The larger pores are consistent with our prior findings, attributed to the phase separation between PDMS and graphite induced by DES. Meanwhile, the smaller pores are a consequence of the loose packing of graphite powder after DES evaporation. As a result, we successfully achieved a hybrid microporous/micropyramid structure through a one-step printing process. To validate the porogen effect of DES, we printed a film using ink without graphite addition, as depicted in Figure S6. The resulting porous PDMS film exhibited pore sizes ranging from 5 to 10 μm. This outcome emphasizes the crucial role of DES in inducing the porous structure, corroborating findings from our previous studies [29]. To validate the template effect of the screen mesh mask, we also printed the film using ink-4 with a stencil mask that lacks a mesh structure. As shown in Figure 2f,g, the composite film produced via stencil printing exhibited a relatively smooth surface, though phase separation and porous structures were still evident (Figure 2g). These results unequivocally confirm the formation mechanism described in Figure 1c. The porous structure is induced by the DES, while the screen mesh induces a micropyramid structure. Thus, we have achieved a complex conductive architecture through a one-step printing and annealing process, facilitated by the ingenious design of the ink and the selection of the fabrication process.

### 3.3. Characterization of Sensing Performance

The electrical–mechanical performance of the printed sensors has been comprehensively characterized and is illustrated in Figure 3. Unfortunately, ink-1 could not form a stable device due to its poor film morphologies. Consequently, the resistance response to pressure for ink-2, ink-3, and ink-4 was compared and is shown in Figure 3a. The presence of the micropyramid structure plays a pivotal role in enhancing the sensor’s sensing range while maintaining a high level of sensitivity. In the case of the sensors based on ink-2 and ink-3, a drastic resistance drop was observed in the small pressure region (<2 kPa), resembling a more switch-like behavior. This characteristic renders these sensors unsuitable for measuring the magnitude of force in certain applications. We attribute this behavior to their higher initial resistance and the rapid increase in contact area between the sensing layer and the electrodes, resulting from the lack of a micropattern surface structure (see Figure S7). In contrast, the sensor based on ink-4 demonstrated excellent pressure sensing performance with both remarkable sensitivity and an extended sensing range. We calculated the sensitivity of the ink-4 sensor using Equation (1):

Sensitivity = ΔR/R_0_ ΔP (1)

where ΔR is the resistance change under the applied pressure, R_0_ is the resistance at the no pressure state, and ΔP is the applied pressure. The sensor demonstrates a three-linear range with a sensitivity of 0.15 kPa^−1^ in the small pressure region (0–2.2 kPa), 0.01 kPa^−1^ in the middle pressure region (2.2–31.8 kPa), and 0.003 kPa^−1^ in the pressure range from 31.8 kPa up to 60 kPa. Furthermore, the sensor exhibits an observable resistance response even for pressures up to 120 kPa (Figure S8).

We also compared the pressure sensing performance of sensors based on different printing methods, as illustrated in Figure 3b. Screen-printed films display superior sensing performance, while stencil-printed films exhibit an on–off behavior due to the absence of the micropatterned surface structure. For sensors without a porous structure, we conducted a performance characterization, as shown in Figure S8. These sensors exhibited pressure responses within a small sensing range. However, upon further force increase, a positive piezoelectric effect was observed. This behavior is attributed to the interaction between the PDMS and graphite within a randomly structured composite, consistent with the results reported in our prior work [29,30]. Hence, the combination of a porous structure and the pyramid structure significantly contributes to the reliability, sensitivity, and expanded sensing range of the sensor. For further characterization, we selected the screen-printed sensor using ink-4 as the optimized sensor. This sensor displayed a rapid response when subjected to a transient force of 0.5 N, with a response time calculated at 116 ms and a recovery time of 178 ms (Figure 3c). Cyclic stability is another critical parameter for sensors. As shown in Figure 3d,e, we subjected the sensor to a cyclic force of 1.5 N for 1000 cycles, and the resistance changes remained consistently stable without significant degradation. These rapid responses and excellent stability undoubtedly underscore the sensor’s potential for real-world applications.

The impact of the outside temperature on sensor sensitivity is crucial for practical applications. We assessed the sensor’s resistance change on a hot plate within the temperature range of 25 °C to 50 °C, applying loading forces of 0 N and 1.5 N, as illustrated in Figure S9. Notably, a decrease in resistance with increasing temperature was observed. We calculated the temperature coefficient of resistance, revealing a value of approximately 2%/°C, likely attributed to the negative temperature coefficient (NTC) effect of carbon materials. Considering the sensor’s high sensitivity and the potential temperature variations in the human body or environmental conditions within wearable devices, we believe that this temperature effect may not significantly impact sensor performance. Furthermore, we observed that, at different temperatures, the sensor’s resistance change in response to pressure remains relatively constant, suggesting that the sensitivity may not be greatly affected. However, careful calibration considerations are essential when developing the circuit for future applications.

Table S2 presents a comprehensive comparison which underscores the distinctive advantages of our novel conductive composite film-based sensor over recently reported resistive-type sensors. The key indicators considered encompass the materials system, device type, fabrication process, as well as the sensitivity and working range. Our sensor demonstrates a notably wide working range compared to micropattern-type sensors; a feature crucial for applications requiring adaptability to varying pressure conditions. Simultaneously, our sensor exhibits high sensitivity, surpassing porous-type sensors. This exceptional combination of attributes highlights the synergistic performance benefits derived from the hybrid porous/micropyramid architecture. Additionally, the use of graphite as the conductive filler gives our sensor cost-effectiveness and accessibility advantages over counterparts employing carbon nanotubes (CNTs), silver nanowires (AgNWs), or graphene oxide (GO). Moreover, the fabrication process, involving a simple ink mixture and one-step screen printing, significantly reduces the complexity inherent in alternative methods such as dip-coating, spray coating, and micromolding. This simplicity enhances scalability and patternability, emphasizing the practicality and efficiency of our fabrication approach.

### 3.4. Application Demonstrations

**Figure 4:**
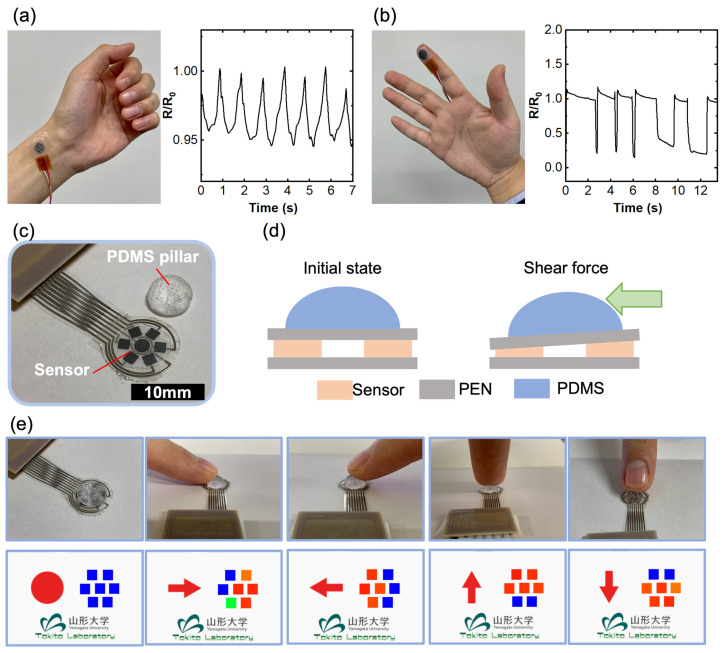
Application demonstrations of the printed sensors. (**a**) A single printed sensor affixed to a human wrist for the measurement of pulse waves. (**b**) A single printed sensor attached to the fingertip for monitoring finger tapping. (**c**) A printed sensor array designed for shear force detection. (**d**) Illustration of the mechanism behind the shear force detection. (**e**) Results of shear force detection facilitated by a GUI system, demonstrating the sensor response as the finger exerts pressure on the PDMS pillar from various directions.

As previously described, the sensor’s ease of fabrication, scalability, and patterning, combined with its high-performance electrical–mechanical detection capabilities, make it a versatile tool with the potential for various applications. Figure 4 presents a proof-of-concept demonstration of our developed sensor. When attached to the human wrist, the sensor exhibits a pronounced resistance change that correlates with the pulse wave, as illustrated in Figure 4a. This response is attributed to the high sensitivity, fast response time, and flexibility of our sensor. It underscores the sensor’s potential for development as a high-performance healthcare device. Additionally, our sensor finds utility in monitoring human hand gestures when affixed to the fingertip, as demonstrated in Figure 4b. The distinct resistance changes observed during different finger tapping provide evidence of the sensor’s applicability for human motion monitoring devices, including applications in augmented reality (AR) and virtual reality (VR).

The sensor’s exceptional patterning capability allows for the creation of high-resolution sensor arrays, with individual sensor units as small as 2 mm by 2 mm. To illustrate this, we designed a seven-sensor array with one sensor in the center and six surrounding sensors for shear force detection. A PDMS pillar was employed to redistribute the applied shear force as a normal force (Figure 4c). The sensing mechanism is elucidated in Figure 4d: when a shear force is applied to the PDMS pillar from one side, an asymmetric normal force is generated due to the PDMS pillar, with one side experiencing a lesser force and the other side encountering a greater force. Consequently, the force direction can be easily detected. By integrating an Arduino and GUI design, we successfully showcase shear force detection, as depicted in Figure 4e and Supporting Video S1. In a theoretical calculation, assuming each sensor provides a 10-bit resolution, resulting in 1024 distinguishable pressure levels, we determined the total number of divisions around the circle by multiplying the number of sensors by the resolution per sensor: 6 × 1024. Subsequently, the angular resolution of the sensor array in degrees was calculated by dividing 360 degrees by the total number of divisions. This calculation yields an angular resolution per division of approximately 0.0586 degrees. This value indicates the system’s capability to distinguish different force directions based on pressure readings from the present sensor array. Furthermore, with the further development of software and algorithms, the magnitude of the force can also be accurately evaluated. These results clearly validate the advantages of our printed sensors in practical applications as multifunctional force sensors, opening up opportunities for a wide range of applications.

## 4. Conclusions

In this study, we have introduced a straightforward and innovative approach for the fabrication of a novel conductive composite film, characterized by a hybrid microporous/micropyramid structure. This development has enabled the creation of high-performance flexible pressure sensors designed to address multifunctional force perception requirements. The construction of complex conductive architectures, such as the combination of microporous and micropatterning structures, has posed a significant challenge in the field of flexible sensors. As a result, a disparity has existed between high-performance sensor capabilities and practical manufacturing processes. In this work, we have pioneered the use of a porous ink in conjunction with screen mesh printing to achieve a hybrid microporous/micropyramid structure through a straightforward, single-step printing and annealing process. This innovative approach eliminates the need for complex and costly procedures that previous research endeavors have often required. Our research provides a fresh perspective on overcoming the challenges associated with high-performance conductive composites, leveraging skillful ink design and process selection. Its significance extends to both academic exploration and industrial utilization, bridging the gap between sensor capabilities and real-world manufacturing processes. Due to constraints in the screen mask structure, this work was limited to producing a micropyramid pattern. However, exploring alternative patterns is feasible by adjusting the printing method, such as utilizing DIW (direct ink writing), thereby opening new avenues for high-performance pressure sensors.

## Data Availability

Data are contained within the article and supplementary materials.

## Supplementary Materials

The following supporting information can be downloaded at: https://www.mdpi.com/article/10.3390/nano14010063/s1, Figure S1: The synthesis process of the printed composite ink; Figure S2: A printed sensing layer with pattern feature of 2 mm by 2 mm; Figure S3: Optical microscopy image of the screen mesh mask and SEM of the printed film with a marked size; Figure S4: Illustration depicting the formation process of the micropyramid structure; Figure S5: SEM images of ink-5 with high loading of graphite; Figure S6: SEM image of printed film without loading of graphite; Figure S7: illustration of contact point changes of different composite films; Figure S8: Comparison of performance of inks with DES (ink-4) and without DES (ink-0). Figure S9: Impact of temperature on the pressure response of the sensor. Table S1: Components of the printable composite inks; Table S2: Comparison of the present pressure sensor with previously reported resistive-type pressure sensors. Video S1: Demonstration of shear force detection using a printed sensor array.
