# Supplementary material for: Printed Composite Film with Microporous/Micropyramid Hybrid Conductive Architecture for Multifunctional Flexible Force Sensors
Source: Nanomaterials (Basel). 2023 Dec 25;14(1):63. doi: 10.3390/nano14010063 (PMC10781003; doi:10.3390/nano14010063)
Supplement: Supplementary file 1 [file nanomaterials-14-00063-s001.zip › nanomaterials-2755345-supplementary.pdf]

Supporting Information

Printed Composite Film with Microporous/Micropyramid Hybrid Conductive Architecture for Multifunctional Flexible Force Sensors

Yi-Fei Wang *, Junya Yoshida, Yasunori Takeda, Ayako Yoshida, Takeru Kaneko, Tomohito Sekine, Daisuke Kumaki and Shizuo Tokito *

Research Center for Organic Electronics (ROEL), Yamagata University, 4-3-16, Jonan, Yonezawa 992-8510, Yamagata, Japan; tww84653@st.yamagata-u.ac.jp (J.Y.); y.takeda@yz.yamagata-u.ac.jp (Y.T.); ayako_yoshida@yz.yamagata-u.ac.jp (A.Y.); t221438m@st.yamagata-u.ac.jp (T.K.); tomohito@yz.yamagata-u.ac.jp (T.S.); d_kumaki@yz.yamagata-u.ac.jp (D.K.)

* Correspondence: wang@yz.yamagata-u.ac.jp (Y.-F.W.); tokito@yz.yamagata-u.ac.jp (S.T.)

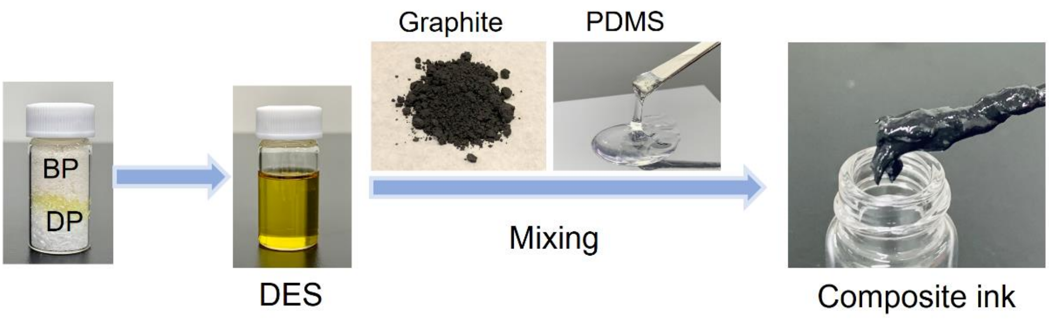

Figure S1. The synthesis process of the printed composite ink

Table S1. Component of the printable composite inks

Inks	PDMS (g)	DES (g)	Graphite (g)
Ink-0	1	0	1.25
Ink-1	1	1	0.5
Ink-2	1	1	0.75
Ink-3	1	1	1.0
Ink-4	1	1	1.25
Ink-5	1	1	1.37

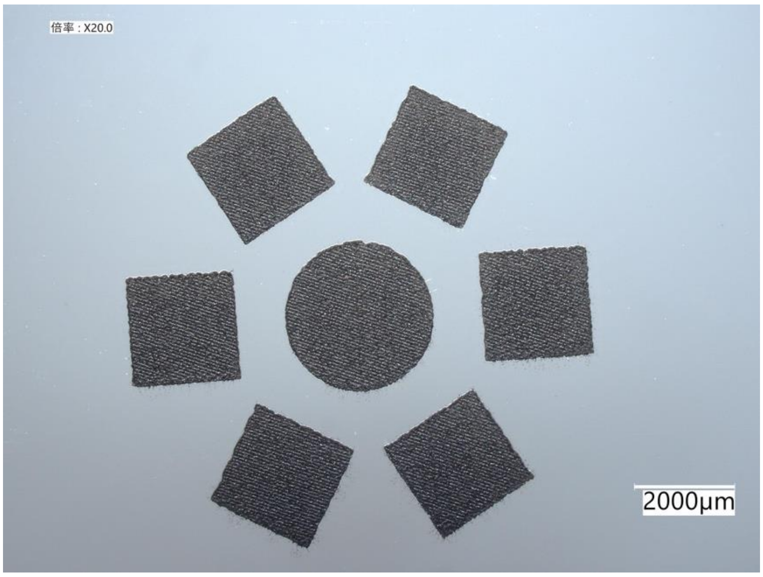

Figure S2. A printed sensing layer with pattern feature of 2 mm by 2 mm.

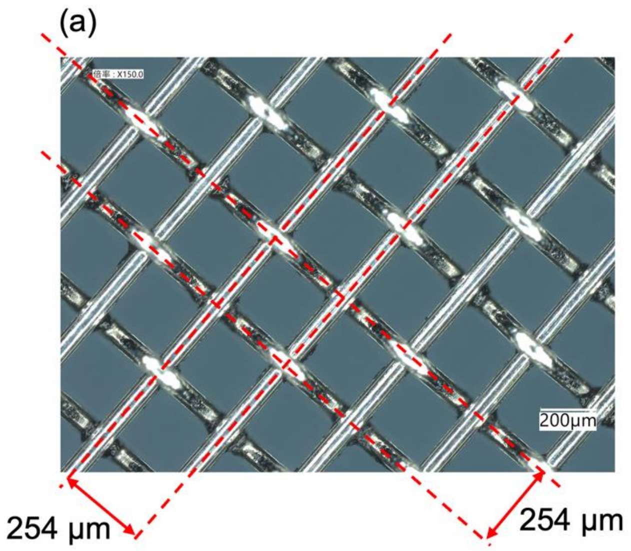

Figure S3. (a) Optical microscopy image of the screen mesh mask. (b) SEM image of the printed composite film. The pitch size (~250 μm) of the micropyramid structure in the composite film perfectly matches the line distance of the screen mesh.

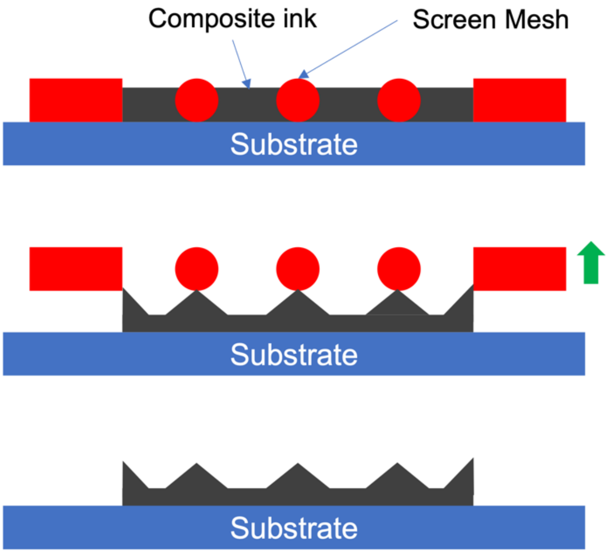

Figure S4. Illustration depicting the formation process of the micropyramid structure in the composite film (ink-4) using a screen mesh.

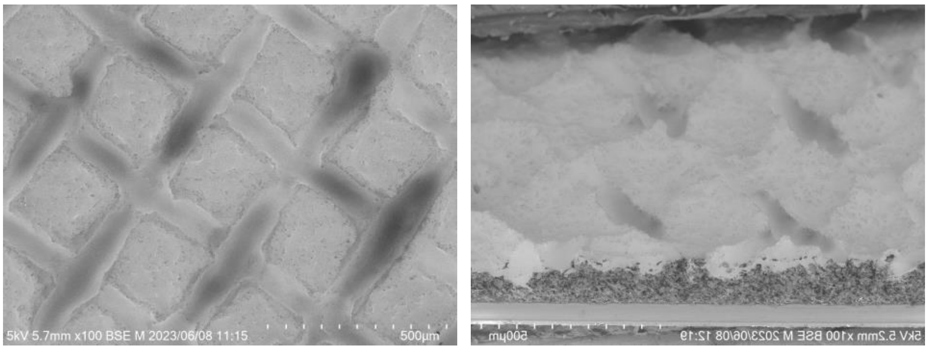

Figure S5. SEM images of Ink-5 with high loading of Graphite taken from the surface (left) and a 45-degree observation angle (right).

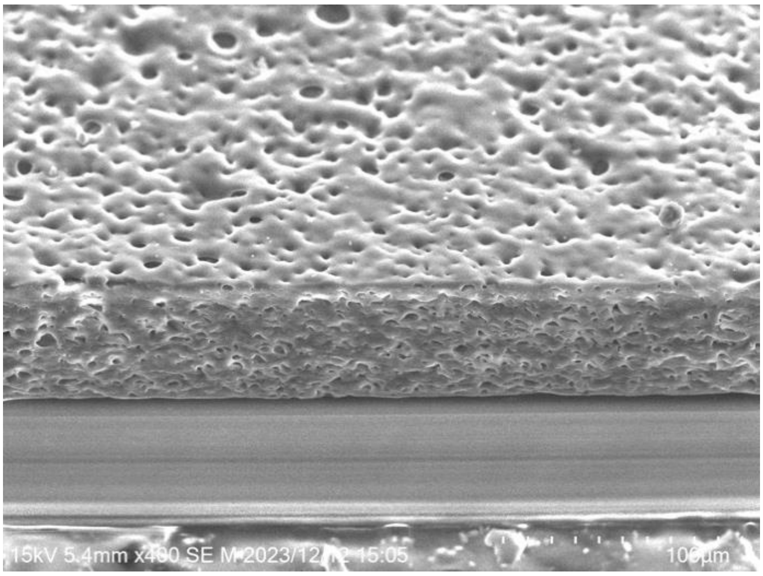

Figure S6. SEM image at a 45-degree observation angle of printed film without loading of graphite.

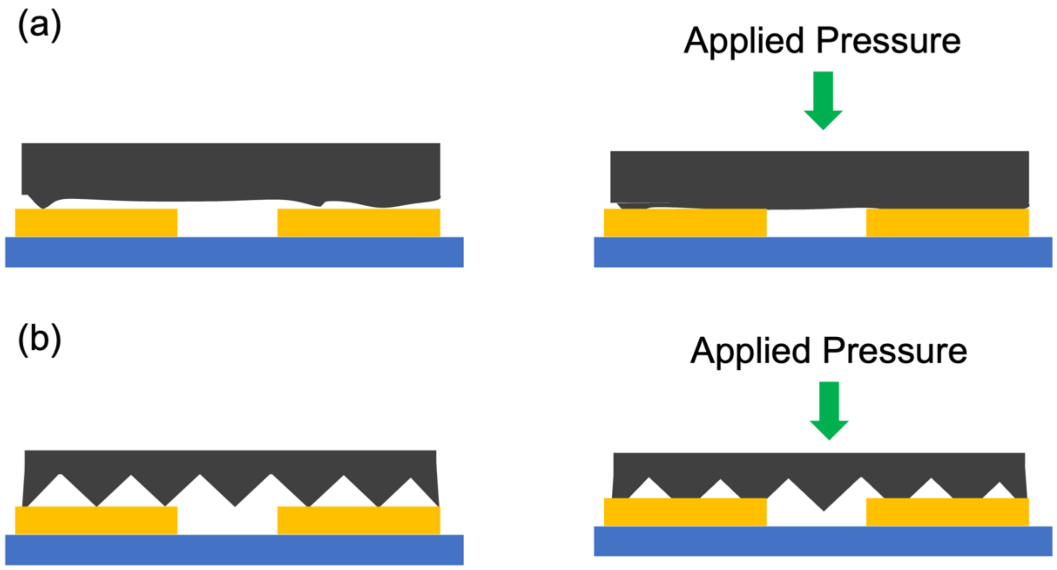

Figure S7: illustration of contact point changes of different composite film. (a) relative flat composite film by ink-2 and ink-3. (b) micropyramid composite film by ink 4. Despite the relatively flat nature of the film, subtle raised structures are present in these printed films, hindering full contact between the film and the electrode. This results in fewer contact points when compared to films with micropyramid surface structures, such as Ink-4. Consequently, sensors based on Ink-2 and Ink-3 exhibit a larger initial resistance. However, when pressure is applied, these films quickly achieve large-area contact between the sensing layer and the electrodes, leading to a rapid change in resistance. This phenomenon explains why Ink-2 and Ink-3 demonstrate greater resistance drop compared to Ink-4 in the small pressure region.

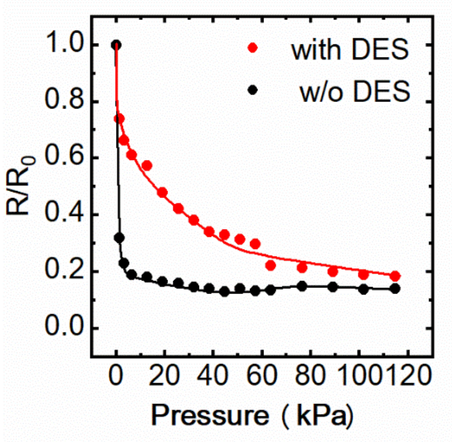

Figure S8. Comparison of performance of inks with DES (ink-4) and without DES (ink-0).

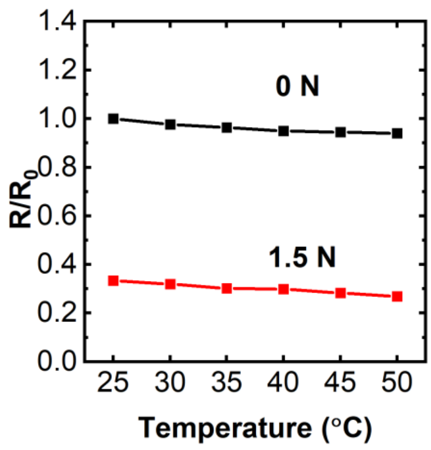

Figure S9. Impact of temperature on the pressure response of the sensor.

Table S2. Comparative analysis of the printed pressure sensor in the present study with previously reported resistive-type pressure sensors.

Materials	Device type	Process	Sensitivity (Work Range)	Reference
PDMS/Graphite	hybrid	1. One step ink mixing<br>2. One step screen-printing<br>3. Device assembly	0.15 kPa^{-1} (0-2.2 kPa)<br>0.01 kPa^{-1} (2.2-31.8 kPa)<br>0.003 kPa^{-1} (31.8-60 kPa)	Our work
TPU/SWCNT	micropattern	1. Spray coating SWCNT on TPU<br>2. Micropatterning TPU on a master mold by hot air blow<br>3. Face to face assembly	0.02 kPa^{-1} (0-20 kPa)<br>0.0007 kPa^{-1} (20-200 kPa)	Chemical engineer journal 407(2021) 127960
GO/sandpaper	micropattern	1. Coating PDMS on sandpaper and peeling<br>2. Coating GO<br>3. GO reduction<br>4. Face to face package	0.25 kPa^{-1} (0-2.6 kPa)<br>0.0045 kPa^{-1} (2.6-40 kPa)	ACS Nano 2018, 12, 3, 2346–2354
TPU/WCNT	micropattern	1. Electrospinning TPU fibrous network<br>2. Dipping in MWCNT solution with sonication<br>3. Device assembly	2 kPa^{-1} (0-0.2 kPa)<br>0.5 kPa^{-1} (0.2-1 kPa)<br>0.02 kPa^{-1} (1-10 kPa)	Composite science and technology 203(2021) 108617
AgNW	micropattern	1. Microwrinkle PDMS master mold by prestretching and UVO treatment<br>2. Coating PDMS and peeling off<br>3. AgNW spray coating<br>4. Device assembly	0.06 kPa^{-1} (0-2 kPa)<br>0.006 kPa^{-1} (2-8 kPa)	Measurement 201(2022)111645
PEDOT:PSS	micropattern	1. making PEDOT:PSS/ graphene hybrid ink<br>2. aerosol jet printing micropattern on IDE electrode<br>3. spray coating MWCNT on sandpaper<br>4. coating ecoflex and peeling off<br>5. assembly device	0.203 kPa^{-1} (1-4 kPa)<br>0.01 kPa^{-1} (4-20 kPa)	J. Mater. Chem. C, 2023,11, 13324-13334

PDDA/MWCNT	micropattern	1. Master mold by photolithography and etcthing.<br>2. Micropattened PDMS by micromolding<br>3. Rinsing in etOH and surface treatment<br>4. Rinse in PDDA and PSS<br>5. 15 times rinsing in PDDA and MWCNTs<br>6. Device assembly	2.65 kPa^{-1} (0-0.3 kPa)	Sensors 2019, 19, 4985
CNT-Coated PDMS sponge	microporous	1)Immerge cubic sugar in PDMS;<br>2) Cut and remove sugar by water to achieve PDMS sponge;<br>3) Immerge PDMS sponge in CNT solution;<br>4) Attach on copper electrode by conductive adhesive	0.02 kPa^{-1} ($P < 100$ kPa)<br>0.01 kPa^{-1} (100-1200 kPa)	ACS Appl. Mater. Interfaces 2019, 11, 23639.
Cross-linked PVDF-HFP/<br><br>fluorosurfactant foam	microporous	1) self-foaming in a glass mold;<br>2) cut and attach on 3D electrode pattern	0.0982 kPa^{-1} ($P < 10$ kPa)<br>0.005 kPa^{-1} (10-20 kPa)<br>0.0025 kPa^{-1} (20-100 kPa)	Nat.<br><br>Commun. 2020, 11, 5747.
PANIH/C-RGO@PU sponge	microporous	1) immerge melamine sponge in GO and reduction;<br>2) immerge in PANI precursors and reaction<br>3) coat conductive paste dot	0.0021 kPa^{-1} (0-2.3 kPa)<br>0.0109 kPa^{-1} (2.3-25 kPa)	Small 2018, 14, 1803018.
Sugar-templated CNT-PDMS sponge	microporous	1) infiltrate PDMS in cubic sugar;<br>2) dissolve sugar by hot water to achieve PDMS sponge;<br>3) repeating drop/dry CNT solution into PDMS sponge<br>4) attach PET/ITO substrate as electrode	0.033 kPa^{-1} (0-15 kPa)<br>0.008 kPa^{-1} (15 kPa-50 kPa)	Small 2017, 13, 1702091.

rGO/Polyaniline Wrapped Sponge	microporous	1) immerge melamine sponge in GO and reduction;<br>2) immerge in PANI precursors and reaction<br>3) smear conductive paste	0.152 kPa^{-1} (0- 3.24 kPa)<br>0.0049 kPa^{-1} (12-27 kPa)	Nanoscale 2018, 10, 10033
